# International Union against Tuberculosis

**Published:** 1922-08

**Authors:** 


### INTERNATIONAL UNION AGAINST TUBERCULOSIS.

THE third International Conference against Tuberculosis was opened on July 11 at the Palais des Académies, Brussels.

Sir Robert Philip gave an address in which he said that the International Union was the direct result of the renewal of the campaign against tuberculosis at the end of the war. The Union was inaugurated in Paris in 1920, and was consolidated in London at the time of the Conference last year. That was not the moment to estimate the place which Belgium held in science, in art, and in commerce. But it was appropriate that, as retiring President of the Union, the speaker should mention the important part played by her in the campaign against tuberculosis. It was a remarkable and significant fact that Belgium—a country in which industrialism was developed to a very high degree, and in which consequently it might be supposed the conditions would be favourable to the incidence of tuberculosis—should have the lowest rate of mortality among civilised peoples. Such facts seemed to prove an exceptionally keen national foresight in sanitary matters, and especially a sane and practical understanding of the causes of tuberculosis and of the measures to take against this scourge. The Belgian Government had set an admirable example in facing its responsibilities so fully, and in voting large sums for tuberculous dispensaries, sanatoria, and other institutions, as well as for public education regarding the disease. Thanks to the foresight of Malvoz and his collaborators, Belgium had been one of the first countries to recognise the great aim and the immense possibilities of the tuberculosis dispensary as the first line of defence and attack against the scourge of civilisation.

### Tuberculosis at School Age.

Dr. Fergus Hewat said that the estimation of the frequency of tuberculosis at school age was vastly important. Sir Robert Philip urged the importance of this 30 years ago in building up his anti-tuberculosis scheme. The school rather than the hospital should be the field of exact inquiry as to tuberculosis in childhood. Twenty years ago Sir Robert Philip found that 30 per cent. of school children presented some evidence of tuberculosis. The seeds of tuberculosis were commonly sown in childhood, and the disease at that age is usually benign and easily treated. Tuberculosis could be eradicated if the sources of infection could be closed down. At present this was not possible. They must search for the earliest manifestations of disease in the infected child, and deal promptly with the condition. The universality of infection and low rate of mortality at school age were apt to lull them into false security. The presence of a positive tuberculin test revealed infection—nothing more and nothing less. It must not be regarded as evidence of aggressive tuberculous disease. On the other hand, its significance must not be overlooked. The school medical officer's position was difficult. He had to regard tuberculosis from the point of view of its interfering with education rather than from the point of view of the early manifestations of infection. Standards of diagnosis differed. Tuberculosis was commonly viewed in too limited a fashion, that was, in respect of particular organs affected, rather than as a spreading disease, with tendency to extension throughout the body.

Considerable misunderstanding existed as to the significance of the commonly employed terms "infection" and "clinical tuberculosis." On the one hand they had the overwhelming proportion of individuals who had been infected with tubercle bacilli, and the relatively small proportion who suffered from tuberculosis disease as evidenced by local manifestations. The fear of the word tuberculosis to the lay mind was still great. The public required to be educated to the view that a timely discovery of infection might by simple means prevent grave trouble later. A slight alteration in the daily life of the child might be quite sufficient to save disaster. By use of the tuberculin test most valuable information might be gained. A positive reaction would suggest a review of environment, a more systematic search for sources of infection, more special consideration of minor diseases, &c., occurring in the subject of the test.

The best results might be obtained by treatment and education running concurrently in children showing early evidence of tuberculosis. This was introduced at the Royal Victoria Hospital, and its value had been subsequently recognised by the Education Authority. The experience at Edinburgh, and in the London Tuberculosis Schools and elsewhere, had been most gratifying. The principle of the open-air school was slowly gaining ground, and as Sir George Newman had said: "In no case has the principle been abandoned after trial." To obtain the best results the fullest co-operation was necessary between the school medical service, the family doctor, the tuberculosis officer, and the Child Welfare Centre.

Much of the benefit derived at open-air day schools might be undone by poor home conditions, and, therefore, facilities for residential schools should, in selected cases, be available. The Grancher system in France had shown valuable results. By using the school rather than the hospital for such scientific investigation of tuberculosis, much value would accrue to individual children, and ways would be further opened for the detection of the sources of infection and conditions of environment, which prejudiced the infected individual's capacity for resistance.

---

### THE BRITISH ASSOCIATION.

The meeting of the British Association will begin at Hull on September 6. Sir Charles Sherrington, the President of the Royal Society, will be the President for the year. The place of the late Dr. W. H. R. Rivers, who was to have been President of the Section on Psychology, will be taken by Dr. C. S. Myers, F.R.S. The Association is making a great effort this year to secure an increased attendance of the younger scientific students. British Association exhibitions have been offered to the Universities and University Colleges, the Senates being asked to nominate students not higher than the B.Sc. degree. The exhibition will give the student his travelling expenses and his membership.

